# scLDM: a conditional diffusion framework for single-cell perturbation prediction

**DOI:** 10.1093/bioinformatics/btag727

**Published:** 2026-09-28

**Authors:** Boyang Wu, Yuhang Liu, Yue Cheng, Xiangrong Liu, Quan Zou, Leyi Wei, Lei Xu

**Affiliations:** School of Information, Xiamen University, Xiamen, 361005, China; Faculty of Applied Sciences, Macao Polytechnic University, Macao SAR, 999078, China; School of Electronic and Communication Engineering, Shenzhen Polytechnic University, Shenzhen, 518055, China; School of Information, Xiamen University, Xiamen, 361005, China; Institute of Fundamental and Frontier Sciences, University of Electronic Science and Technology of China, Chengdu, 611731, China; Faculty of Applied Sciences, Macao Polytechnic University, Macao SAR, 999078, China; School of Electronic and Communication Engineering, Shenzhen Polytechnic University, Shenzhen, 518055, China

## Abstract

**Motivation:**

Accurate prediction of single-cell responses to external stimuli is pivotal for deciphering gene regulatory mechanisms and accelerating data-driven drug discovery. However, effectively capturing the complex, non-linear mapping between intrinsic cell states and external stimuli remains an open problem.

**Results:**

We propose scLDM, a generative framework based on latent diffusion models for predicting single-cell perturbation responses. scLDM first compresses high-dimensional gene expression into a compact latent space via a variational autoencoder, followed by a conditional diffusion process to generate post-perturbation states, explicitly guided by pre-perturbation cellular state, cell type, and perturbation type. Systematic evaluations on six datasets across diverse biological settings, spanning pharmacological stimulation, viral infection, helminth infection, genetic perturbations, and multi-species immune response contexts, demonstrate that scLDM achieves superior predictive accuracy compared to state-of-the-art methods. Furthermore, the model exhibits strong interpretability, as the learned perturbation embeddings show high functional alignment with known biological mechanisms. Overall, scLDM provides a robust and biologically consistent strategy for in silico perturbation screening.

**Availability:**

The code is available at https://github.com/samrogers1233/scLDM and archived on Zenodo at https://doi.org/10.5281/zenodo.22658164.

## 1 Introduction

Cellular perturbation serves as the fundamental engine for constructing virtual cells, enabling the mechanistic dissection and *in silico* simulation of biological systems (Ji *et al.* 2021, Bunne *et al.* 2024, Dimitrov *et al.* 2026). By intentionally altering cellular states through exogenous or endogenous interventions, researchers can probe the causal dependencies of life (Peidli *et al.* 2024). These perturbations span diverse chemical and genetic types, including drug treatments, gene knockouts, and CRISPR-based gene editing (Wei *et al.* 2025). In the context of single-cell omics, coupling perturbation experiments with high-throughput technologies such as scRNA-seq enables researchers to characterize adaptive cellular changes, infer gene regulatory mechanisms, and identify key signaling pathways (Replogle *et al.* 2020). Furthermore, these approaches reveal causal relationships between genotypes and phenotypes (Kolodziejczyk *et al.* 2015, Wilson *et al.* 2015, Svensson *et al.* 2017). While such advances provide a theoretical foundation for precision medicine and drug discovery (Chang *et al.* 2024), the prohibitive cost and scalability limits of experimental screening pose substantial barriers (Luecken and Theis 2019, Wang *et al.* 2023). Consequently, developing computational approaches to predict cellular perturbation responses *in silico* has emerged as a critical strategy.

Given the limitations of traditional machine learnings in handling sparse data and modeling nonlinear perturbation effects (Wei *et al.* 2026), a variety of deep learning-based approaches have been proposed (Qi *et al.* 2024, Roohani *et al.* 2024, Liu *et al.* 2025, Shang *et al.* 2025, He *et al.* 2026). Among representative methods, scGen (Lotfollahi *et al.* 2019) leverages the latent space of variational autoencoders (VAEs) (Kingma and Welling 2014) to implement vector arithmetic, effectively assuming a linear model of perturbation responses. However, by approximating perturbation effects using the mean response across different cell types, scGen overlooks heterogeneity in perturbation responses between cell types. Building upon this, scPRAM (Jiang *et al.* 2024) incorporates attention mechanisms (Vaswani *et al.* 2017) and optimal transport (OT) to better account for single-cell level heterogeneity. Yet, it still primarily models the transition from control to perturbed states as a latent displacement, often simplifying complex, non-linear biological processes into linear shifts. Similarly, CPA (Lotfollahi *et al.* 2023) predicts combinatorial effects by linearly combining disentangled representations of perturbations and covariates. While effective for conditional predictions, this heuristic strategy struggles to capture cell-specific uncertainty. Beyond VAE-based approaches, scPreGAN (Wei *et al.* 2022) employs generative adversarial networks (GANs) (Goodfellow *et al.* 2020) to predict perturbation responses. However, GAN-based modeling is inherently difficult for single-cell data, due to the training process is highly unstable and prone to mode collapse. Consequently, the generated data often suffer from limited diversity and a loss of essential biological semantics.

Most recent methods primarily model perturbation responses by maximizing data likelihood in latent space (Viñas Torné *et al.* 2026). However, reliance on restrictive priors often leads to oversmoothed generations, causing a loss of fine-grained biological detail. Moreover, these approaches typically fail to fully leverage the intrinsic state features of perturbed cells to effectively guide the prediction process. Fundamentally, generative mechanisms built on fixed distributional assumptions struggle to capture the pervasive cell-to-cell heterogeneity and stochasticity inherent in single-cell data, limiting their ability to represent the true distribution of perturbation responses. In contrast, denoising diffusion probabilistic models (Ho *et al.* 2020) approximate complex data distributions through a forward diffusion process and a learnable reverse denoising process. This paradigm enables high-fidelity, diverse generation with improved generalization (Liu *et al.* 2025). Their relatively stable training dynamics and robustness to noise highlight substantial potential for single-cell data modeling. Given the high-dimensional and sparse nature of single-cell transcriptomic data, adapting this approach via latent diffusion models (Rombach *et al.* 2022) offers a computationally efficient and theoretically robust alternative.

Here, we propose scLDM, a generative framework that synergizes a variational autoencoder (VAE) with a latent diffusion model (LDM) to predict single-cell perturbation responses. The framework is trained through a three-stage process. First, a VAE is employed to map high-dimensional, sparse gene expression profiles into a compact latent space, effectively extracting robust cellular representations. Second, to address the lack of cell-cell correspondence inherent in destructive single-cell sequencing, we utilize optimal transport (OT) to infer trajectory couplings, constructing a paired training dataset that links control states to their perturbed counterparts in the latent space. Third, leveraging these OT-aligned pairs, we train a conditional diffusion model to predict the post-perturbation latent representation. Crucially, this generation process is guided by pre-perturbation latent state and specific conditions, such as cell type and perturbation type. Finally, the predicted latent representations are decoded back into the original gene expression space via the VAE decoder. By incorporating unified compositional perturbation embeddings, scLDM supports the modeling of both single and combinatorial responses, enabling the prediction of unseen combinatorial perturbations. We evaluated scLDM on six public datasets spanning drug stimulation, genetic perturbations, and multi-species immune response modeling, demonstrating its superior performance.

## 2 Methods and materials

### 2.1 Overview of the scLDM framework

The scLDM framework synergizes a variational autoencoder (VAE) with a conditional latent diffusion model (LDM) to predict single-cell perturbation responses. Formally, given a high-dimensional gene expression profile $x\in R^{G}$, the VAE encoder maps both control and perturbed cells into a shared compressed latent space z=E(x), ensuring that biological variations are captured in a unified manifold (Fig. 1a). By minimizing the transport cost between distributions, OT aligns cellular states across conditions, thereby constructing paired training data $\left(z_{c},z_{p}\right)$ in the latent space (Fig. 1b). Because this latent space serves as the basis for both diffusion modeling and optimal-transport-based cell matching, we further evaluated the VAE reconstruction quality on the PBMC validation set. The VAE achieved strong reconstruction performance, and the original and reconstructed cells showed consistent structure in joint UMAP visualization (Table S1 and Fig. S1). Leveraging these aligned pairs, the core of scLDM is trained to learn the conditional distribution $p(z_{p}|z_{c},c,p)$. The training involves a forward process that progressively adds Gaussian noise to $z_{\mathrm{post}}$, and a reverse denoising process where the model learns to recover the original state (Fig. 1c). Here, the generation of the target latent state $z_{p}$ is strictly guided by the initial control state $z_{c}$, along with specific biological conditions including cell type c and perturbation type p.

**Figure 1:**
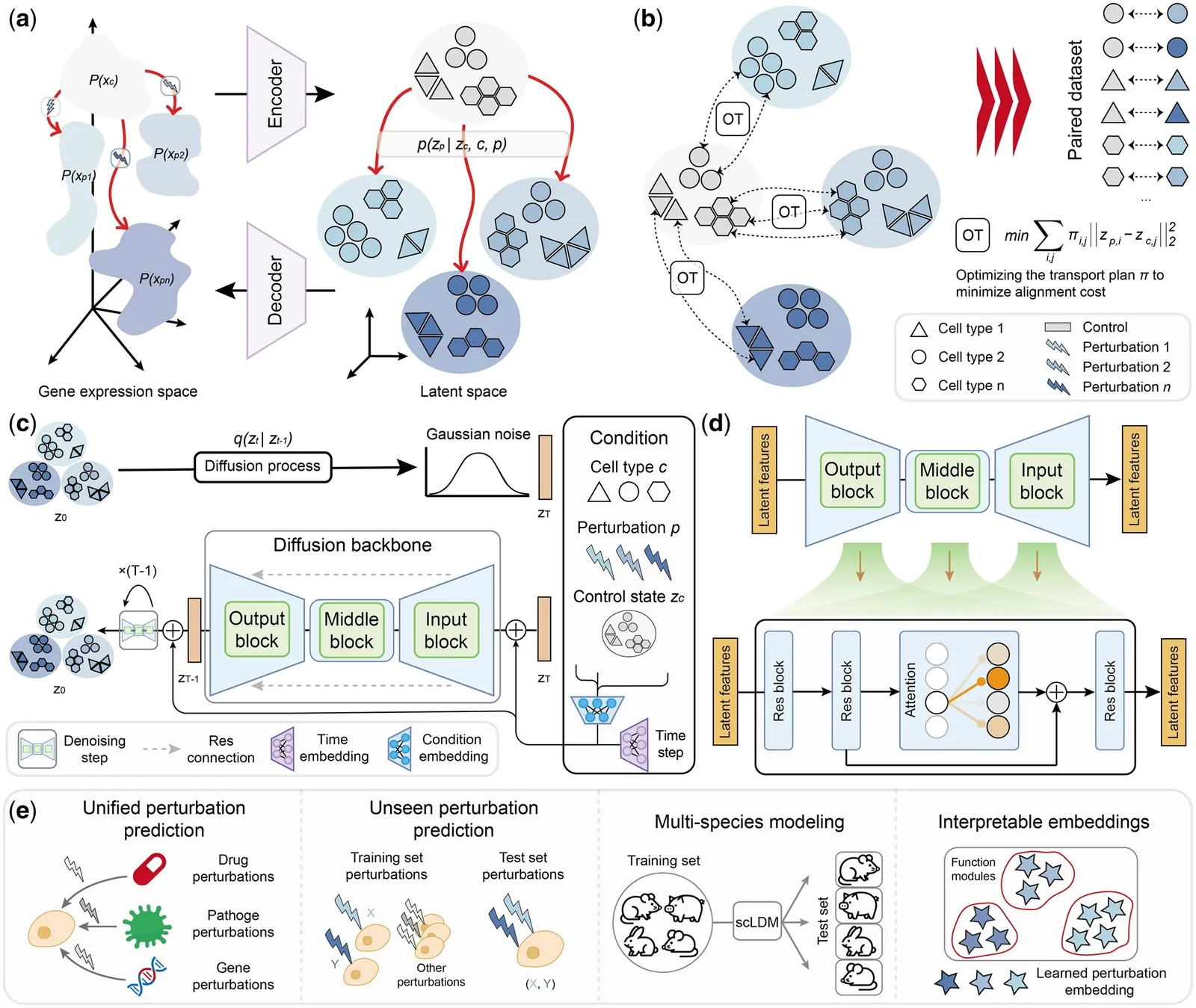
Overview of the scLDM framework for single-cell perturbation predictions. (a) VAE projects high-dimensional gene expression profiles of both pre- and post-perturbation cells into a shared low-dimensional latent space to capture biological manifolds. (b) Cell alignment via optimal transport. In the latent space, the OT algorithm is employed to infer probabilistic couplings between unpaired pre- and post-perturbation data. (c) Conditional diffusion architecture. The framework involves a forward process that gradually adds Gaussian noise to latent states and a reverse generative process. The denoising network takes the noisy state as input, with control states, perturbation types, and cell types injected as conditioning signals to guide the reconstruction. (d) An attention mechanism is incorporated to dynamically integrate conditioning contexts with latent features. (e) scLDM facilitates a wide range of perturbation analysis tasks.

Architecturally, the denoising process is governed by a conditional denoising network designed to integrate these complex conditions (Fig. 1d). The backbone utilizes a U-Net-like structure comprising input, middle, and output blocks. These blocks share a consistent modular design, each incorporating residual blocks and attention mechanisms. This hierarchical design allows the model to effectively fuse the conditioning signals into the latent feature extraction process, ensuring that the predicted shifts in cell state are both biologically context-aware and mathematically robust.

During the inference phase, scLDM enables the prediction of cellular responses to various perturbation scenarios (Fig. 1e). Starting from random Gaussian noise, the model performs iterative denoising to generate the target latent representation, which is subsequently decoded to reconstruct full gene expression profiles. Leveraging compositional perturbation embeddings, scLDM extends beyond reproducing observed data, demonstrating the capacity to generalize to previously unseen combinatorial perturbations.

### 2.2 Variational autoencoder

To address the high dimensionality and sparsity of single-cell transcriptomic data, scLDM utilizes a VAE to learn a continuous latent representation. For a single cell, let $x\in R^{G}$ denote its gene expression vector over G genes, and let $z\in R^{d}$ denote its d-dimensional latent representation. The encoder $q_{\phi }(z|x)$, parameterized by ϕ, maps x into the latent space, whereas the decoder likelihood $p_{\theta }(x|z)$, parameterized by θ, reconstructs x from z. We assume a standard normal prior p(z)=N(0,I), where I is the d×d identity matrix. Because the true posterior $p_{\theta }(z|x)$ is intractable, we introduce an approximate posterior $q_{\phi }(z|x)$ to approximate it, yielding the following decomposition:


(1) log ⁡pθ(x)=KL(qϕ(z|x)||pθ(z|x))+L(θ,ϕ;x)

where KL(⋅∥⋅)≥0, the evidence lower bound (ELBO) provides an optimizable objective:


(2) log ⁡pθ(x)≥L(θ,ϕ;x)

where,


(3) L(θ,ϕ;x)=Eqϕ(z|x)[logpθ(x|z)]-DKL(qϕ(z|x)||pθ(z))

where $D_{\mathrm{KL}}(\cdot ∥\cdot )$ denotes the Kullback–Leibler divergence, $E_{q_{\phi }(z|x)}[\cdot ]$ denotes the expectation with respect to the approximate posterior $q_{\phi }(z|x)$, and L(θ,ϕ;x) denotes the evidence lower bound.

Maximizing the marginal log-likelihood is therefore achieved by maximizing the ELBO, which encourages accurate reconstruction of the original data distribution while regularizing the latent space to match the prescribed standard normal prior. Accordingly, the VAE loss is defined as:


(4) LVAE=-Eqϕ(z|x)[logpθ(x|z)]+αKL(qϕ(z|x)||pθ(z))#

where the first term promotes reconstruction quality, the second term enforces the latent prior, and α controls their relative weighting.

During optimization, the VAE samples latent variables from the approximate posterior:


(5) qϕ(z|x)=N(z|μϕ(x),σϕ2(x)I)#

While maintaining gradient backpropagation. Using the reparameterization trick,


(6) z=μϕ(x)+σϕ(x)⋅ϵ, ϵ∼N(0,I)#

where $\mu_{\phi }(x)\in R^{d}$ and $\sigma_{\phi }(x)\in R_{+}^{d}$ denote the mean and element-wise standard deviation predicted by the encoder, respectively, and ϵ∼N(0,I) denotes a standard Gaussian noise vector. Through this procedure, the VAE embeds both pre-perturbation and post-perturbation cellular expression profiles into a unified latent space, providing a structured representation for the subsequent diffusion model to learn perturbation-induced responses. We further evaluated the VAE reconstruction quality on the PBMC validation set and reported the reconstruction errors, mean expression PCC, and joint UMAP visualization in Supplementary Note S1, Table S1, and Fig. S1.

### 2.3 Latent diffusion model for perturbation prediction

scLDM leverages a conditional latent diffusion model (LDM) to probabilistically model perturbation responses. By explicitly conditioning on pre-perturbation states, cell types, and perturbation types, the model effectively captures cellular heterogeneity. The training involves a forward diffusion process where Gaussian noise is progressively added to the VAE-encoded post-perturbation latent vectors, generating noisy targets for the denoising network. Let $z_{p}^{0}$ denote the clean post-perturbation latent representation and $z_{p}^{t}$ denote its noisy version at diffusion step t, where t∈{1,…,T} and T is the total number of diffusion steps. The superscript t indicates the diffusion time step, and the subscript p indicates the perturbed state. The forward transition distribution $q(z_{p}^{t}|z_{p}^{t-1})$ is defined as:


(7) q(zpt|zpt−1)=N(zpt|1-βtzpt−1,βtI)#

where $\beta_{t}\in (0,1)$ is the variance schedule parameter that increases with the time step.

Let $z_{c}^{0}$ and $z_{p}^{0}$ denote the clean latent representations of the control and perturbed cells, respectively, where the subscripts c and p indicate the control and perturbed states. Let c denote the combined conditioning vector containing the cell-type and perturbation-type embeddings. scLDM models the conditional distribution $p_{\theta }(z_{p}^{0}|z_{c}^{0},c)$. At diffusion step t, the reverse transition is defined as:


(8) pθ(zpt−1|zpt,zc0,c)=N(zpt−1|μθ(zpt,zc0,c,t),β^tI)#

where θ denotes the parameters of the denoising network, $\mu_{\theta }(z_{p}^{t},z_{c}^{0},c,t)$ is the predicted mean of the reverse transition, and ${\tilde{\beta }}_{t}$ denotes its variance. We further define $\alpha_{t}=1-\beta_{t}$ and $\bar{\alpha_{t}}=\prod_{s=1}^{t}\alpha_{s}$. The noise-prediction network is denoted by $\epsilon_{\theta }(z_{p}^{t},z_{c}^{0},c,t)$:


(9) μθ(zpt,zc0,c,t)=1αtzpt-βt1-α¯tϵθ(zpt,zc0,c,t)#

During training, a diffusion step t∼Uniform{1,…,T} and a Gaussian noise vector ϵ∼N(0,I) are randomly sampled. The denoising network is trained to predict the added noise by minimizing the following mean squared error:


(10) Lsimple(θ)=Ezp0,ϵt,tϵθ(zpt,zc0,c,t)-ϵt2#

The denoising network employs a U-Net-like structure specifically designed for single-cell latent representations. It features a sequence of residual and attention blocks that iteratively refine the latent state, guided by injected conditional embeddings. During inference, the model starts from standard Gaussian noise $z_{T}∼N(0,I)$ and progressively denoises under conditional guidance to generate $z_{p}^{0}$, which is then decoded by the VAE decoder into the post-perturbation gene expression profile. To account for the stochasticity inherent in diffusion sampling, we performed multiple independent sampling runs for each test condition. Specifically, for each condition, the model weights, input control cells, and perturbation conditions were held fixed, while only the random seed used during diffusion sampling was varied. In each run, the post-perturbation latent representations were independently generated and subsequently decoded into predicted gene expression profiles, after which the corresponding evaluation metrics were calculated separately. The quantitative results reported in both the main manuscript and the Supplementary Materials represent the averages across multiple independent sampling runs. For visualization, we present the mean predicted expression profiles obtained across repeated runs, thereby reducing fluctuations arising from any single stochastic sample.

### 2.4 Optimal transport

To align pre-perturbation and post-perturbation cells in the latent space, we adopt the Kantorovich optimal transport framework (Flamary *et al.* 2021). Given the destructive nature of single-cell sequencing, we infer cell correspondences by minimizing the total transport cost required to map the control distribution to the perturbed distribution. The optimization objective is to find a transport plan π that minimizes the aggregate distance:


(11) min∑i,jπij‖zp,i-zc,j‖22#

where $z_{p,i}$ and $z_{c,j}\;$represent the latent vectors of perturbed and control cells respectively, and $\pi_{ij}$ denotes the transport probability between them subject to uniform marginal constraints. To establish deterministic cell pairs for conditional diffusion modeling, we convert the continuous transport plan into hard matches by selecting the control cell with the maximal transport probability for each perturbed cell:


(12) Pair(i)=arg⁡maxj⁡(πij)#

This process effectively identifies the most likely pre-perturbation state for each perturbed cell, thereby constructing a paired dataset that preserves the geometric structure of the biological manifold in the latent space.

### 2.5 Gene perturbation embeddings

To guide the generation process with specific perturbation information, we construct vectorized representations explicitly informed by biological priors using Gene2Vec (Du *et al.* 2019). These 200-dimensional embeddings (Yang *et al.* 2022), which capture functional gene semantics, serve as the initial input to a trainable feed-forward perturbation encoder.

Specifically, for single-gene perturbations, we directly retrieve the corresponding vector of the target gene. For multi-gene perturbations, we aggregate the representations by computing the element-wise mean of the vectors for all involved genes. This design effectively integrates co-expression-derived priors into the perturbation space, thereby enhancing the model’s ability to discriminate between diverse perturbation types and generalize to complex combinatorial regimes.

### 2.6 Datasets and data processing

To systematically evaluate the generalization performance of scLDM across diverse perturbation types, cell lineages, and species contexts, we curated six benchmark datasets spanning pharmacological stimulation, viral infection, helminth infection, multi-species immune response modeling, and genetic editing. For drug and pathogen-induced responses, we utilized the PBMC dataset (Kang *et al.* 2018), comprising 18,868 human peripheral blood mononuclear cells stimulated with interferon-β (IFN-β) obtained from the GEO database (GSE96583) and preprocessed following scGen protocols. Additionally, we included the Hpoly.Day10 dataset (Haber *et al.* 2017) (GSE92332) to model the transcriptional response of intestinal epithelial cells ten days post-infection with the parasitic helminth Heligmosomoides polygyrus, consisting of 2,711 infected and 3,240 control cells. To capture virus-induced perturbations across heterogeneous tissue microenvironments, we analyzed the COVID-19 dataset processed by Lotfollahi *et al.* (2022), which includes 11,742 SARS-CoV-2 infection and matched control cells across lung, PBMC, and bone marrow tissues. To evaluate performance on genetic perturbations, we employed two Perturb-seq benchmarks based on the K562 leukemia cell line. The Adamson dataset (Adamson *et al.* 2016) profiles transcriptomic changes resulting from 86 distinct single-gene perturbations across 68,599 cells. The Norman dataset (Norman *et al.* 2019) encompasses a more complex perturbation regime, featuring 105 single-gene and 131 combinatorial double-gene perturbations across 105,822 cells, thereby allowing assessment of the model on higher-order genetic interactions. To evaluate multi-species joint modeling of immune responses, we utilized the Species dataset (Hagai *et al.* 2018), which profiles 69,330 single-cell transcriptomes of macrophages from four distinct species (mouse, rat, rabbit, and pig) collected six hours following lipopolysaccharide (LPS) stimulation.

The data processing procedure follows that of Luo *et al.* (2026) For all in-distribution experiments, cells were randomly divided into training, validation, and test sets using an 80%, 10%, 10% split. For the out-of-distribution combinatorial perturbation experiment on the Norman dataset, a fully held-out evaluation protocol was adopted. Each designated double-gene perturbation condition was excluded from model training and was used only for testing. Unless otherwise specified, all experiments were evaluated using 10 independent runs with random seeds “42–51.” Across runs only the source of sampling randomness was changed. For diffusion-based prediction, different seeds generated independent initial Gaussian noise for the reverse-diffusion process. For VAE reconstruction, latent representations were independently sampled through the reparameterization procedure. Evaluation metrics were calculated separately for each run and summarized as the mean and standard deviation across the 10 runs. Expression-based visualizations were generated using the element-wise mean of the 10 resulting expression matrices.

### 2.7 Implementation details

The VAE maps each input gene-expression profile into a 100-dimensional latent representation. The VAE was trained using the AdamW optimizer with a learning rate of $1\times 10^{-3}$, a weight decay of $1\times 10^{-4}$, and a KL-divergence loss weight of $5\times 10^{-4}$. During VAE training, Gaussian noise with a scale of 0.1 was added to the normalized expression input.

The diffusion process operates in the low-dimensional latent space. The 100-dimensional noisy latent variable is first projected into a 512-dimensional hidden representation and is then processed by a U-shaped residual architecture with hidden dimensions of 512, 256, and 128. The decoding path symmetrically integrates intermediate representations through skip connections and maps the resulting representation back to the 100-dimensional latent space. Each residual block primarily consists of normalization, SiLU activation, and linear transformations. Diffusion-timestep information and control-state information are projected into a conditioning embedding and incorporated into the residual blocks through scale-and-shift modulation. Self-attention is applied to selected intermediate and bottleneck representations to model dependencies among latent features. The resulting denoising network contains approximately 3.08 million trainable parameters.

The forward diffusion process uses 1000 diffusion steps with a linear noise schedule. During training, a diffusion timestep is randomly sampled, and the denoising network is optimized using a mean-squared-error objective to predict the Gaussian noise added to the latent variable. The diffusion model uses the AdamW optimizer with a learning rate of $1\times 10^{-4}$ and a weight decay of $1\times 10^{-4}$. An exponential moving average of the model parameters is maintained during training with a decay rate of 0.9999. All models were trained on a single NVIDIA RTX 4090 GPU.

## 3 Results

### 3.1 scLDM accurately predicts transcriptional responses across diverse biological systems

To benchmark scLDM across diverse biological systems, we conducted comprehensive experiments on three representative datasets: PBMC (drug stimulation), COVID-19 (viral infection), and H. poly (parasitic infection). We compared scLDM against three state-of-the-art baselines, including scGen, scPRAM, and scPreGAN.

We first assessed the consistency between the predicted and ground-truth transcriptional profiles using the squared pearson correlation coefficient ($R^{2}$). As shown in Fig. 2a, scLDM achieved the highest $R^{2}$ values across most cell types in all three datasets, outperforming the baselines. To rigorously test the model’s ability to capture biologically significant signals, we focused our evaluation on the top 100 differentially expressed genes (DEGs) identified between control and perturbed conditions (Wolf *et al.* 2018). In the correlation analysis of these DEGs (Fig. 2a), scLDM demonstrated a tighter alignment with the ground truth compared to other models. This indicates that scLDM not only preserves the global expression structure but also accurately recapitulates the regulatory patterns of key genes driving the perturbation response.

**Figure 2:**
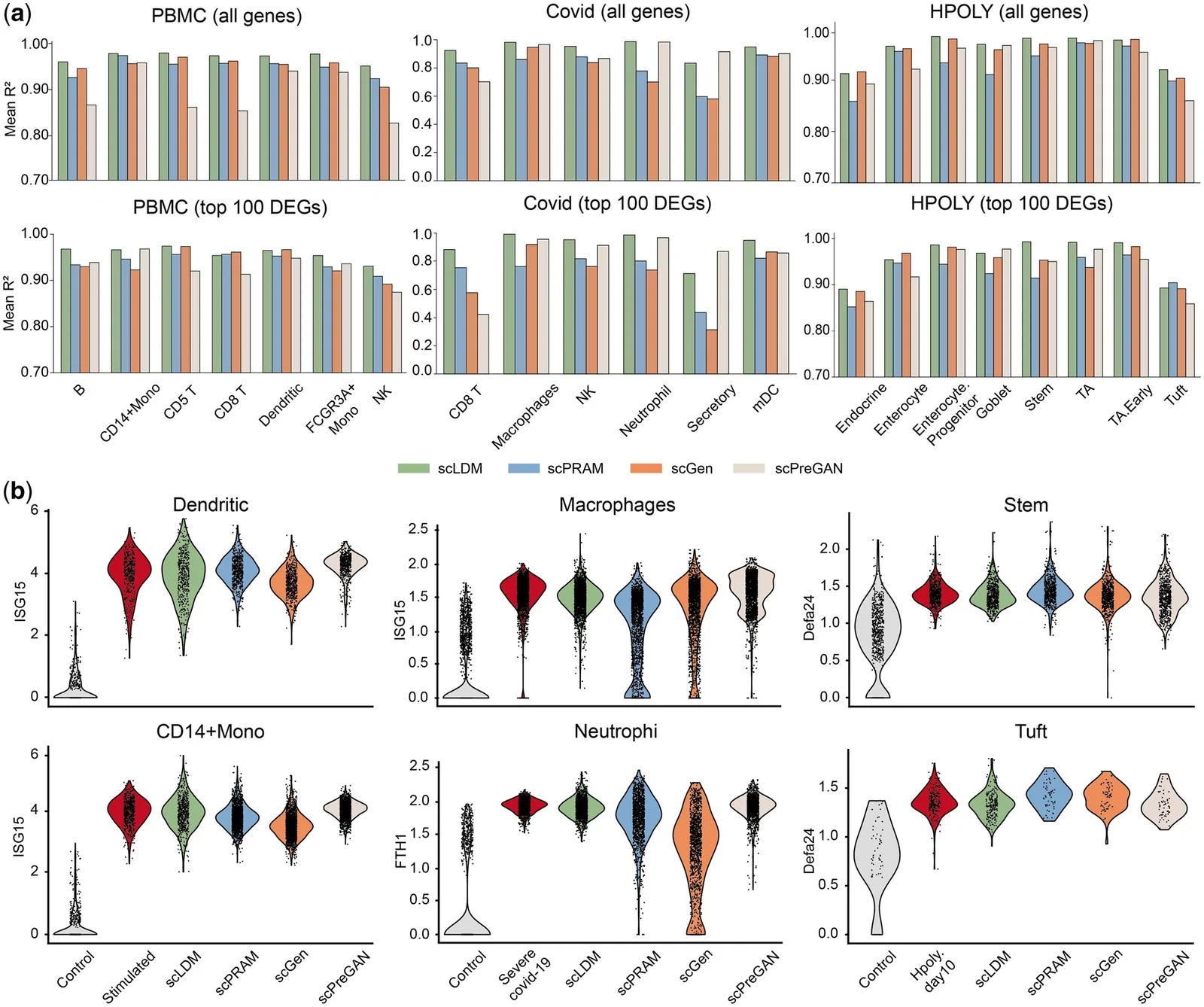
Accurate prediction across diverse perturbation settings. Results are shown for three benchmark datasets: PBMC IFN-β stimulation, COVID-19 infection, and H. polygyrus infection. (a) Performance metrics comparing predicted and ground-truth expression profiles across three datasets, summarized as the mean across 10 independent runs. Top: Metrics calculated using all genes. Bottom: Metrics calculated using the top 100 DEGs. (b) Violin plots displaying the expression distributions of representative top-ranked DEGs across different cell types. Distributions compare ground truth with predictions from scLDM and baseline methods.

Beyond mean expression values, capturing the full distributional shape of gene expression is critical for analyzing perturbation intensity and heterogeneity. We selected representative DEGs from two distinct cell types in each dataset to inspect their expression distributions (Fig. 2b). The distributions generated by scLDM closely matched the ground-truth densities. In contrast, baseline models often failed to capture these nuances, leading to over-smoothed or shifted distributions. This confirms that scLDM accurately reconstructs perturbation effects at both the population average and single-gene resolution.

To quantify the distributional fidelity of the predictions, we calculated the Wasserstein distance (Panaretos and Zemel 2019) between the predicted and real expression profiles for the top 100 DEGs. As shown in Fig. 3a, scLDM yielded the lowest median Wasserstein distances across all datasets. This result confirms that our model minimizes the discrepancy between predicted and actual perturbation states, thereby offering a more precise characterization of expression shifts than competing methods.

**Figure 3:**
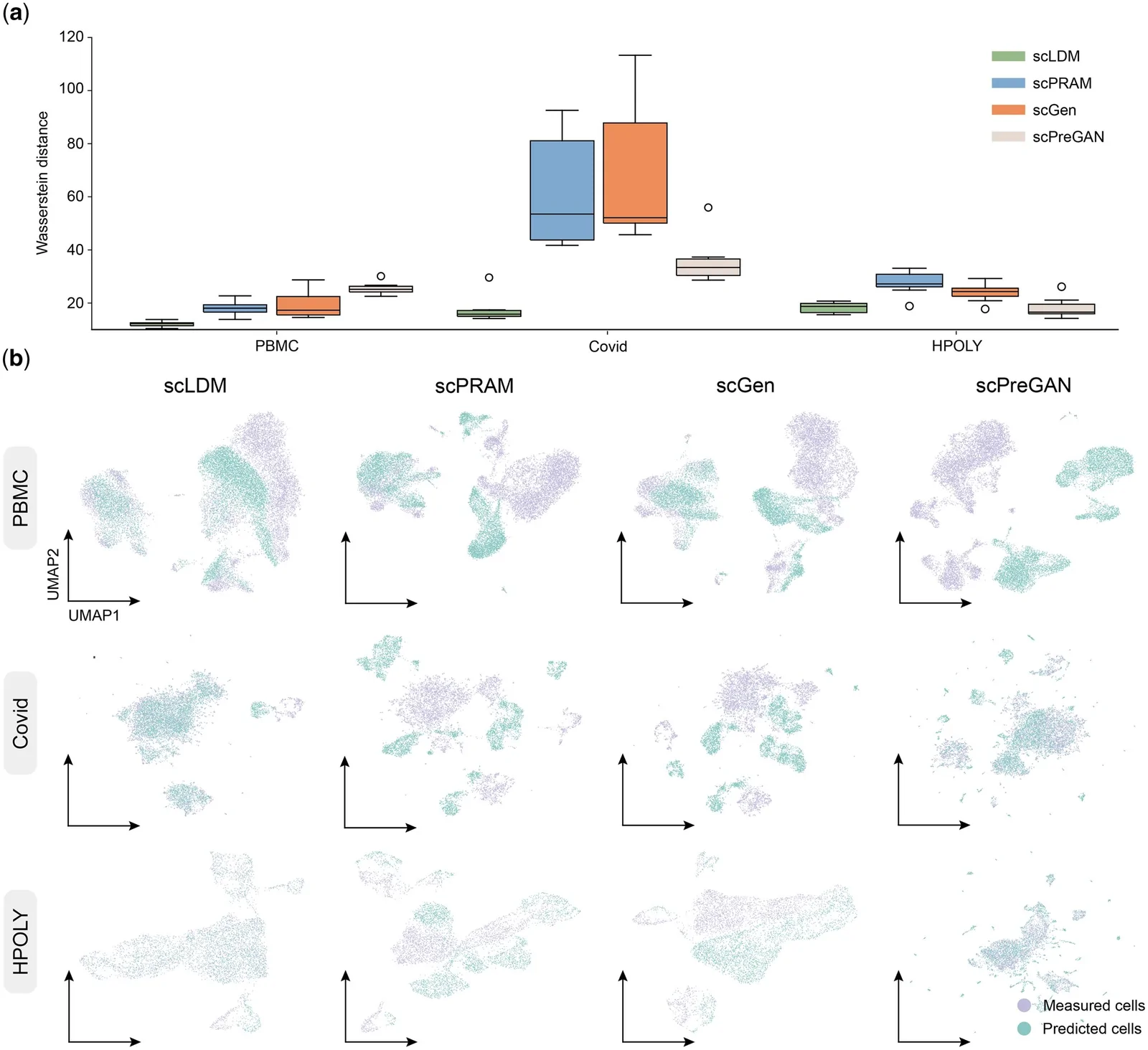
scLDM minimizes distributional shifts and preserves manifold topology. Results are shown for PBMC IFN-β stimulation, COVID-19 infection, and H. polygyrus infection. (a) Box plots showing Wasserstein distances between the predicted and ground-truth distributions of the top 100 DEGs. Center lines represent medians; box limits indicate the 25th and 75th percentiles; whiskers extend to 1.5-times the interquartile. (b) UMAP visualizations overlaid with ground-truth and predicted cells.

Finally, to visualize the overall structural of the predicted cells, we merged the predicted data with the ground truth and performed dimensionality reduction using UMAP visualization (Fig. 3b). In the low-dimensional embedding space, cells generated by scLDM exhibited overlap with the true perturbed cells, suggesting a successful reconstruction of the underlying data manifold. Conversely, baseline methods showed varying degrees of deviation. These results demonstrate that scLDM offers superior structural fidelity and robustness in modeling complex perturbation responses.

### 3.2 scLDM captures complex outcomes of single and combinatorial genetic perturbations

We next evaluated the performance of scLDM in predicting transcriptional responses to genetic perturbations of varying complexity, ranging from single-gene perturbations to combinatorial perturbations.

First, we evaluated the performance of single-gene perturbations using the Adamson dataset, which profiles single-gene perturbations of 86 genes in K562 cells. We benchmarked scLDM against CPA and scGen. Quantitatively, we assessed reconstruction fidelity using the squared correlation coefficient on the top 3,000 highly variable genes (HVGs). As shown in Fig. 4a, scLDM achieved $R^{2}$ values exceeding 0.9 for the majority of perturbation conditions. In comparison, CPA and scGen exhibited wider variance, with distributions concentrated primarily in the 0.7–0.9 range. To capture distributional shifts in pathway-relevant signals, we further calculated the Wasserstein distance for the top 100 DEGs across 10 randomly sampled perturbations (Fig. 4b). scLDM yielded the lowest average distances, indicating a precise alignment with the ground-truth distributions of key regulatory genes.

**Figure 4:**
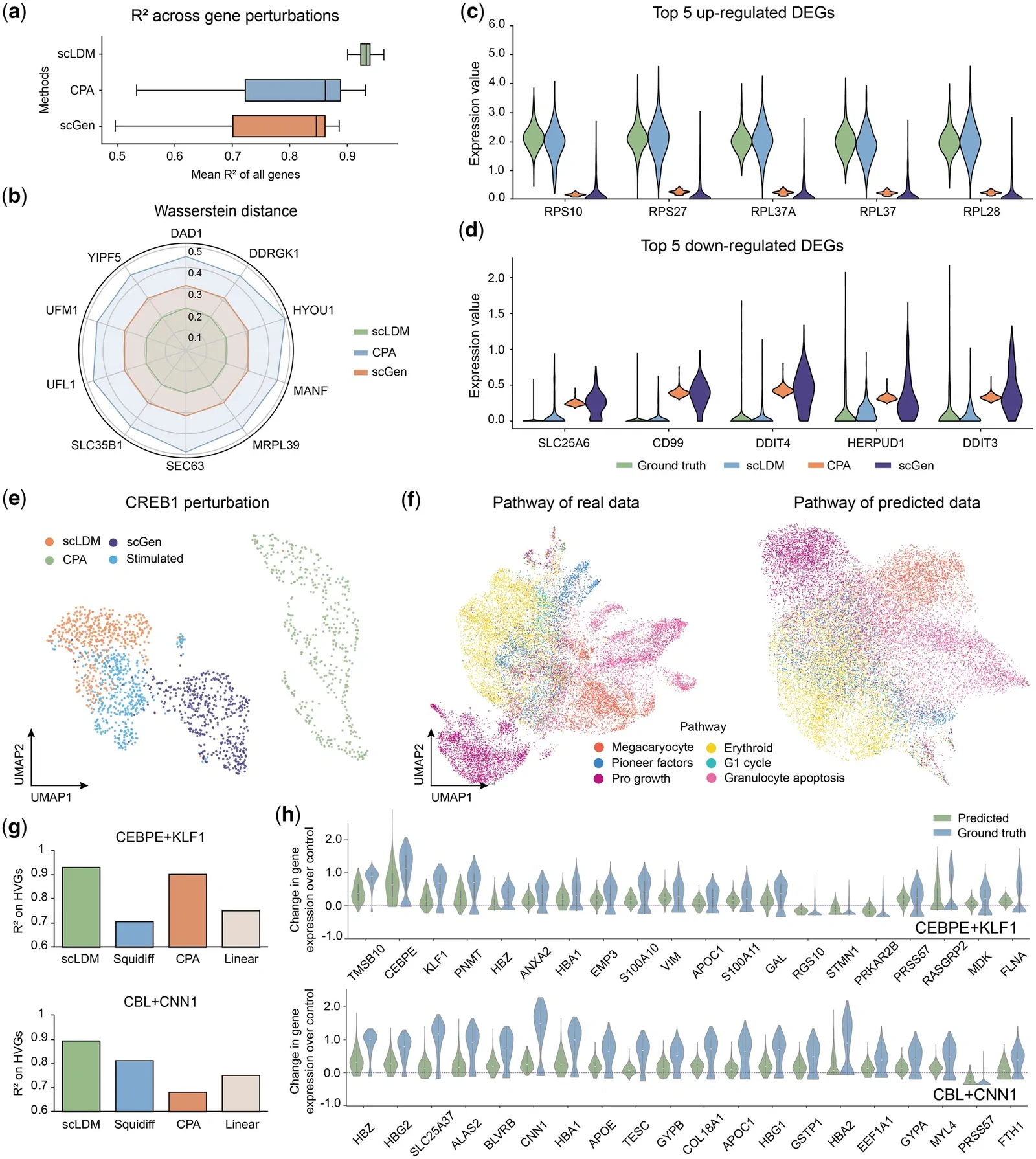
Robust prediction of genetic perturbation responses and generalization to unseen combinations. Results are based on the Adamson Perturb-seq dataset for single-gene perturbation prediction and the Norman Perturb-seq dataset for combinatorial perturbation prediction. (a) Performance benchmarking across genetic perturbation tasks. Box plots display the median (center line), the interquartile, and whiskers extending to 1.5-times the interquartile. (b) Average Wasserstein distances for 10 perturbation conditions randomly sampled from 86 scenarios. (c, d) Distributions of the top five upregulated (c) and downregulated (d) genes under CREB1 perturbation. (e) UMAP visualization of predicted and ground-truth cells under CREB1 perturbation. (f) UMAP visualizations of generated post-perturbation profiles. Cells are colored by functional modules. (g) Mean $R^{2}$ on HVGs for the unseen combinatorial perturbation CEBPE+KLF1 and CBL+CNN1. (h) Expression distributions of the top 20 DEGs for the combinatorial perturbations CEBPE+KLF1 and CBL+CNN1.

We further performed a detailed analysis using CREB1 perturbation as a representative case study, as CREB1 is a transcriptional regulator with clear perturbation-associated DEG responses in the Adamson dataset. Visualizing the latent manifold via UMAP (Fig. 4e) reveals that the perturbed cells generated by scLDM closely overlap with the true cell distribution, whereas baselines show varying degrees of manifold shift. At the gene level (Fig. 4c and d), scLDM reproduced both the directionality and the distributional shapes of the top DEGs associated with CREB1 perturbation. In contrast, baseline models exhibited incorrect directional predictions or distributional mismatches, suggesting limitations in capturing specific downstream regulatory effects.

Moving beyond single targets, combinatorial perturbations are critical for understanding genetic interactions and overcoming therapeutic resistance, yet the combinatorial space is often too vast to screen exhaustively. We used the Norman dataset, which contains 237 conditions including both single-gene and two-gene perturbations, to evaluate scLDM in this complex setting.

We examined the biological coherence of the learned latent representations. By projecting the predicted transcriptomes into a UMAP embedding (Fig. 4f) and coloring them by functional modules, we observed that scLDM organizes perturbations into distinct clusters. Functionally related perturbations formed compact groups, suggesting that the model captures intrinsic biological features associated with cell fate.

A critical challenge is predicting the effects of unseen combinatorial perturbations. To evaluate this ability, we performed experiments on 10 fully held-out double-gene perturbations in the Norman dataset, including CEBPE+KLF1, CBL+CNN1, DUSP9 + ETS2, MAP2K3 + MAP2K6, UBASH3B + UBASH3A, SET+KLF1, PTPN12 + PTPN9, ETS2 + CNN1, CEBPE+RUNX1T1, and TBX3 + TBX2. For each test condition, the corresponding double-gene perturbation was excluded from training, allowing evaluation of 0/2 unseen combinatorial perturbation prediction. Across these held-out combinations, scLDM maintained stable prediction performance in terms of $R^{2}$ on HVGs, mean expression PCC, delta PCC on HVGs, and DEG PCC. We further compared scLDM with CPA, a linear additive baseline, and the diffusion-based method Squidiff. Overall, scLDM achieved comparable performance across the main evaluation metrics, with details provided in Supplementary Note S2, Table S2 and Figs. S2–S4.

We next used the perturbation combination CEBPE+KLF1 and CBL+CNN1 as representative fully held-out cases for detailed analysis. Figure 4g presents the reconstruction accuracy for this unseen condition. scLDM outperformed the linear baseline, indicating its ability to capture non-linear genetic interactions that simple additive models miss. Furthermore, we analyzed the expression trends of the top 20 DEGs for CEBPE+KLF1 and CBL+CNN1 (Fig. 4h). In both scenarios, scLDM reflected the diverse up- and downregulation patterns observed in the ground truth, demonstrating its robustness in modeling complex genetic interactions even for unseen combinations.

To further assess whether the predicted perturbation profiles preserve biologically meaningful downstream responses, we compared the differential expression signatures obtained from true perturbation versus control cells with those obtained from predicted perturbation versus control cells. We then performed Gene Ontology Biological Process (GO BP) enrichment analysis using the top 100 DEGs identified from true and predicted perturbation responses for each held-out combinatorial condition (Supplementary Note S3). The GO BP enrichment results provide an additional downstream evaluation of biological fidelity beyond expression-level statistical metrics. Detailed results are provided in Supplementary Fig. S5.

### 3.3 scLDM models perturbation responses in a multi-species setting

Characterizing perturbation responses across species is important for disentangling conserved immune programs from species-specific regulatory mechanisms (Geirsdóttir *et al.* 2019), thereby supporting the translation of findings from animal models to humans. To evaluate the ability of scLDM to predict transcriptomic responses while accounting for inter-species heterogeneity, we analyzed single-cell macrophage responses to LPS stimulation across four distinct species (mouse, rat, rabbit, and pig) (Hagai *et al.* 2018). Adopting an in-distribution random split, we performed joint modeling to evaluate the capacity of scLDM to generate perturbation responses in a multi-species context, benchmarking its performance against scGen, scPRAM, and scPreGAN.

To quantify the overall accuracy of response prediction and the extent to which each method captures species-specific biology, we identified the top 100 DEGs for each species. We then calculated $R^{2}$ between the true and predicted responses at both the mean and variance levels of gene expression. As illustrated in Fig. 5a, scLDM achieved the highest $R^{2}$ values for both mean and variance metrics. The model demonstrated a particularly pronounced advantage in predicting expression variance, indicating that scLDM not only accurately recapitulates general perturbation trends but also captures differences in response magnitude and expression variability across species.

**Figure 5:**
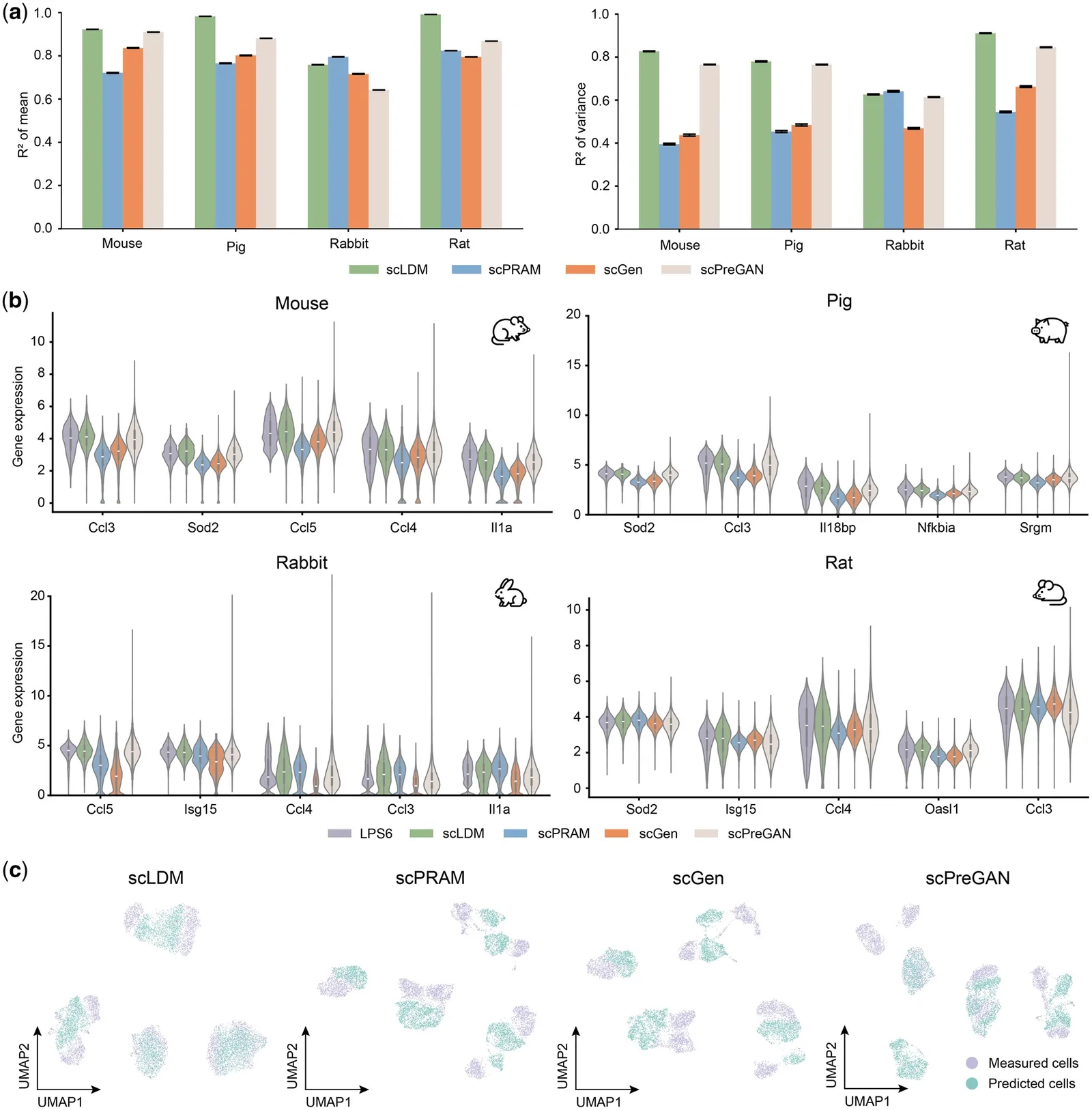
scLDM enables accurate prediction of perturbation responses in a multi-species setting. Results are based on the cross-species LPS stimulation dataset containing macrophages from mouse, rat, rabbit, and pig. (a) Quantitative evaluation of predictive performance, showing the mean and variance of R² across species. Error bars represent the standard deviation across 10 independent runs. (b) Expression distributions of the top five DEGs across species, comparing ground-truth and predicted profiles. (c) Joint UMAP visualizations of predicted post-perturbation cells overlaid with ground-truth perturbed cells.

We further examined the performance of these predictions at the single-gene level by comparing violin distributions of the top five DEGs for each species (Fig. 5b). Across all species and corresponding marker genes, scLDM produced expression distributions that most closely matched the true experimental data. The model recovered both the directionality of regulation and the overall distributional shapes observed in the ground truth. Conversely, baseline methods frequently exhibited systematic shifts in expression levels or insufficient distributional fidelity. Finally, to assess generative performance at the dataset level, we performed a joint UMAP visualization of predicted and true perturbed cells (Fig. 5c). In the multi-species embedding, predictions generated by scLDM overlapped with the true perturbed data, whereas other methods showed deviations in either global structure or the positioning of local clusters. Overall, these results demonstrate that scLDM provides superior predictive performance under multi-species LPS perturbation settings and effectively inter-species heterogeneity.

### 3.4 scLDM learns biologically structured perturbation embeddings

To guide the generation of desired post-perturbation gene expression profiles, scLDM incorporates a gene embedding vector as conditioning information within the latent space. To investigate whether scLDM captures biologically meaningful structures within these representations, we analyzed their latent representations using the Norman combinatorial perturbation dataset. We first constructed initial representations for each perturbation condition based on Gene2Vec (Du *et al.* 2019). These vectors were then processed by the perturbation encoding network of scLDM to generate the final embeddings. After retaining unique embeddings per perturbation type, we performed UMAP dimensionality reduction and colored the resulting projection by functional modules defined by Norman *et al.* (2019) based on post-perturbation transcriptomic similarity.

As shown in Fig. 6, compared to the initial representations, the learned perturbation embeddings exhibit more pronounced functional stratification in the low-dimensional space. Perturbations belonging to the same module aggregate into tighter clusters, while distinct modules such as Erythroid, Pro-growth, and Pioneer factors are well separated. These observations indicate that the encoding process amplifies manifold structures consistent with known gene programs, thereby providing an informative prior that guides the generative modeling along biologically plausible directions.

**Figure 6:**
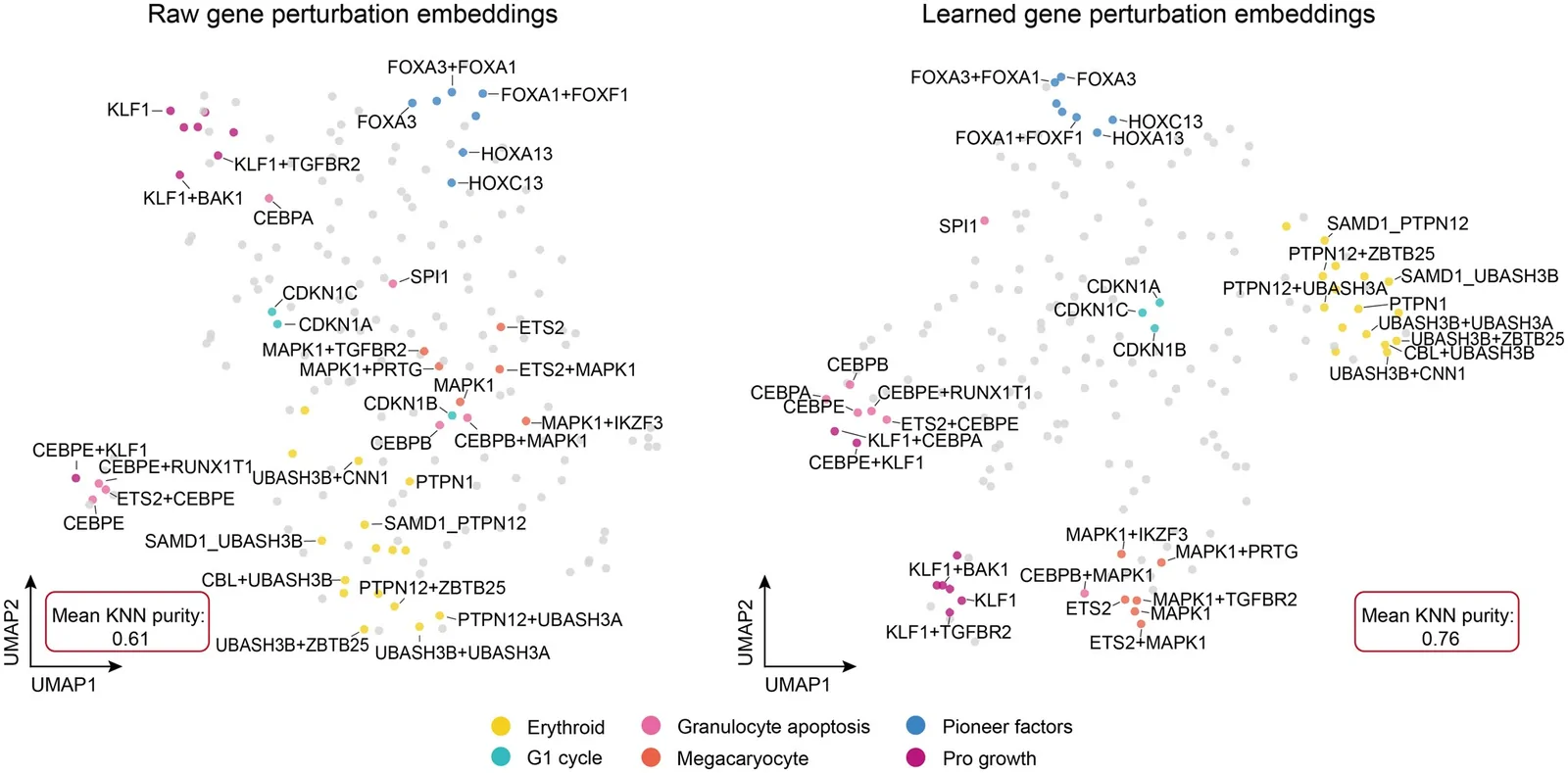
Functional alignment of learned perturbation embeddings. Results are based on perturbation embeddings derived from the Norman dataset. UMAP visualization of raw gene perturbation embeddings, and scLDM’s perturbation embeddings, colored by functional modules.

To quantify cluster compactness, we calculated the neighborhood (KNN) purity for each embedding by determining the proportion of samples from the same module among its k nearest neighbors (k=10). The mean neighborhood purity of the learned embeddings increased by approximately 25% relative to the initial Gene2Vec embeddings. Notably, the embeddings for certain combinatorial perturbations were positioned in proximity to clusters of functionally related single-gene perturbations. This suggests that the model integrates the relationships between single-gene and combinatorial effects into its internal representation rather than merely memorizing discrete labels.

Overall, these results demonstrate that the perturbation embeddings learned by scLDM exhibit a structured organization in the latent space consistent with known perturbation cluster labels. This structured representation facilitates coherent modeling across perturbation types and provides a rationale for the generalization capability of the model regarding unseen combinations.

### 3.5 Ablation study

To quantify the contribution of individual components to predictive performance, we conducted ablation studies. We focused on four key modules: the pre-perturbation cell-state embedding network (CtrlCond), the cell-type embedding network (LabelCond), optimal transport alignment (OT), and the gene embedding network (GeneCond). Using the full scLDM model as a baseline, we evaluated performance changes in ablated variants via $R^{2}$ on all genes and top 100 DEGs, alongside the Wasserstein distance to assess distributional matching (Table 1).

**Table 1:** Ablation analysis of model components.

Settings	$R^{2}$ **(all genes) ↑**	$R^{2}$ **(top 100 DEGs) ↑**	Wasserstein distance ↓
w/o CtrlCond	0.941 ± 0.074	0.943 ± 0.013	14.000 ± 1.069
w/o LabelCond	0.948 ± 0.016	0.950 ± 0.018	13.114 ± 1.080
w/o OT	0.950 ± 0.017	0.951 ± 0.014	13.010 ± 0.907
scLDM (PBMC)	**0.967 ± 0.011**	**0.961 ± 0.014**	**12.073 ± 1.137**
w/o GeneCond	0.833 ± 0.058	0.691 ± 0.080	22.904 ± 3.944
scLDM (Adamson)	**0.929 ± 0.018**	**0.792 ± 0.096**	**21.312 ± 1.690**

Results are reported as the mean and standard deviation over 10 independent runs.

On the PBMC dataset, the removal of CtrlCond resulted in the most significant performance degradation. The $R^{2}$ on all genes decreased from 0.967 to 0.941, and the Wasserstein distance increased markedly from 12.073 to 14.000. This underscores the critical role of pre-perturbation cell-state information in guiding individualized response generation. Similarly, the exclusion of LabelCond or OT led to consistent performance declines across both metrics. These results confirm that cell-type priors are essential for capturing population-specific characteristics and that the state-alignment mechanism is necessary for learning accurate perturbation-induced shifts in the latent space.

For the Adamson dataset, given its focus on genetic perturbations, we examined the contribution of the GeneCond module. The results indicate that eliminating the gene embedding weakens the model’s ability to recognize and generate target perturbation effects. Specifically, $R^{2}$ across all genes fell from 0.929 to 0.833, while accuracy on the top 100 DEGs dropped from 0.792 to 0.691. These findings highlight GeneCond as a decisive factor for learning perturbation-specific expression changes and generating the correct target distributions associated with specific gene targets. Overall, these analyses demonstrate that each component of scLDM is indispensable for achieving high performance and preserving biological structure.

## 4 Discussion

scLDM integrates compressed latent representations with conditional diffusion modeling to capture the non-linear dynamics of single-cell perturbation responses. Unlike methods that rely on direct mappings, our framework learns transformations between cellular states through a progressive denoising generative process. This strategy allows scLDM to explain expression variability associated with diverse biological contexts, facilitating its application across pharmacological stimulation, viral and helminth infections, genetic perturbations, and multi-species perturbation response modeling.

Benchmarking demonstrates that scLDM not only predicts average gene expression changes but also preserves intercellular heterogeneity. This capability is particularly observed in single-gene perturbation and multi-species joint modeling tasks, where the model captures distributional shifts consistent with underlying biological processes rather than merely fitting average effects. Furthermore, the model learns structured latent embeddings that align closely with known gene functional modules. This organized latent space provides a biologically informed representation for the generalization performance of scLDM, especially when predicting responses to previously unseen combinatorial perturbations.

Despite these advances, the current implementation focuses on discrete perturbation conditions and does not account for continuous covariates such as drug dosage or time-series dynamics (Xu *et al.* 2024). Furthermore, predictive performance remains bounded by the biological subspace covered by the training data, meaning that extrapolation to conditions significantly outside the training distribution poses challenges (Adduri *et al.* 2025). In addition, prediction accuracy may vary across perturbation conditions. Performance can be less stable for conditions with limited training support, weak perturbation-induced transcriptional shifts, high sparsity or technical dropout in single-cell gene expression measurements, and combinatorial perturbations involving complex genetic interactions. These cases represent potential failure modes and suggest that future extensions should incorporate larger perturbation panels, uncertainty estimation, and stronger priors for rare or weak-response perturbations. Nevertheless, this work highlights the utility of conditional diffusion modeling in the latent space for perturbation response prediction. Looking forward, we envision scaling scLDM by substituting the current VAE backbone with large-scale pre-trained single-cell foundation models (Szałata *et al.* 2024). By leveraging richer semantic representations from massive cell atlases and extending training to atlas-level perturbation datasets (Zhang *et al.* 2025), this framework can evolve into a general-purpose virtual cell foundation model.

## Supplementary Material

btag727_Supplementary_Data

## Data Availability

All datasets analysed in this study were obtained from publicly accessible repositories. The PBMC dataset is available from the NCBI Gene Expression Omnibus (GEO) under accession number GSE96583, and the Hpoly.Day10 dataset is available under accession number GSE92332. The COVID-19 dataset is available under accession number GSE145926. The Adamson and Norman Perturb-seq datasets are available under accession numbers GSE90546 and GSE133344, respectively. The multi-species macrophage dataset is available from ArrayExpress under accession number E-MTAB-6754.
