# Supplementary material for: scLDM: a conditional diffusion framework for single-cell perturbation prediction
Source: Bioinformatics. 2026 Sep 28;42(10):btag727. doi: 10.1093/bioinformatics/btag727 (PMC13661255; doi:10.1093/bioinformatics/btag727)
Supplement: Supplementary file 1 [file btag727_supplementary_data.docx]

**Supplementary Materials for**

**scLDM: A conditional diffusion framework for single-cell**

**perturbation prediction**

Boyang Wu^1#^, Yuhang Liu ^2#^, Yue Cheng^3^, Xiangrong Liu^1^, Quan Zou^4^, Leyi Wei^2^ and Lei Xu^3*^

^1^ School of Information, Xiamen University, Xiamen, China

^2^ Faculty of Applied Sciences, Macao Polytechnic University, Macao, 999078, SAR China

^3^ School of Electronic and Communication Engineering, Shenzhen Polytechnic University, Shenzhen 518055, China

^4^ Institute of Fundamental and Frontier Sciences, University of Electronic Science and Technology of China, Chengdu, 611731, China

^#^ These authors contributed equally: Boyang Wu and Yuhang Liu

*** Correspondence(s):**

Lei Xu: [csleixu@szpu.edu.cn](mailto:csleixu@szpu.edu.cn)

**Supplementary Results**

**Supplementary Note S1. Evaluation of VAE reconstruction quality**

Because the latent representation learned by the VAE is used as the input space for both latent diffusion modeling and optimal-transport-based cell matching, we quantitatively evaluated the reconstruction quality of the trained VAE. Specifically, we applied the trained VAE to the PBMC validation set and compared the reconstructed expression profiles with the original input expression profiles. Reconstruction quality was evaluated using mean squared error (MSE), root mean squared error (RMSE), mean absolute error (MAE), and mean expression Pearson correlation coefficient (mean expression PCC). Metrics were calculated using all genes and the top 100 differentially expressed genes (DEGs).

Across all validation cells and all genes, the VAE achieved MSE = 0.0097, RMSE = 0.0985, MAE = 0.0237, and mean expression PCC = 0.9856. When evaluated on the top 100 DEGs, the VAE achieved MSE = 0.0283, RMSE = 0.1682, MAE = 0.0848, and mean expression PCC = 0.9975. These results indicate that the VAE effectively preserves the global structure of the input expression profiles and maintains strong consistency for perturbation-associated genes.

To further visualize the consistency between the original and reconstructed expression profiles, we jointly embedded original cells and VAE-reconstructed cells using UMAP. The reconstructed cells largely overlapped with their corresponding original cells in the low-dimensional space, supporting that the learned latent representation retains the major transcriptional structure of the input data.

**Supplementary Note S2. Complete out-of-distribution evaluation of combinatorial perturbations**

To comprehensively evaluate the generalization ability of scLDM to unseen gene combinations, we conducted out-of-distribution experiments on 10 fully held-out double-gene perturbations from the Norman Perturb-seq dataset. For each experiment, the corresponding double-gene perturbation was completely excluded from the training set and used only for testing. We compared scLDM with Squidiff, CPA, and a linear additive baseline. Performance was assessed using R^2^ on highly variable genes (HVGs), mean expression PCC, delta PCC on HVGs, and DEG PCC. The mean performance and standard deviation across the 10 held-out perturbation conditions are summarized in Supplementary Table S2, with the corresponding visualization results presented in Supplementary Figs. S2–S5.

**Supplementary Note S3. Downstream GO Biological Process enrichment analysis**

To evaluate whether the predicted perturbation profiles preserve biologically meaningful downstream responses, we performed Gene Ontology Biological Process (GO BP) enrichment analysis on the held-out combinatorial perturbation task in the Norman Perturb-seq dataset. For each held-out double-gene perturbation, we compared true perturbation cells with control cells and predicted perturbation cells with control cells, respectively. Differentially expressed genes were ranked for each comparison, and the top 100 DEGs were used for GO BP enrichment analysis.

For each held-out perturbation condition, we provide GO BP enrichment bubble plots for true perturbation responses, predicted perturbation responses, and side-by-side comparisons between true and predicted responses. In these bubble plots, the color indicates −log10(FDR), and the dot size indicates the number of genes associated with each enriched GO BP term.

Overall, the predicted perturbation profiles recovered GO BP terms that were broadly consistent with those enriched from true perturbation responses, suggesting that scLDM preserves downstream biological processes associated with combinatorial perturbations beyond expression-level statistical similarity.

**Supplementary Figures**

**Supplementary Figure. S1.**

**Supplementary Figure S1.Joint UMAP visualization of original and VAE-reconstructed PBMC expression profiles.** Original cells and reconstructed cells were jointly embedded based on their gene expression profiles. The overlap between original and reconstructed cells indicates that the trained VAE preserves the major transcriptional structure of the input data.

**Supplementary Figure. S2.**

**Supplementary Fig. S2. Overall comparison of R² on HVGs across models.** Bars show the mean R² on HVGs across 10 fully held-out double-gene perturbations in the Norman dataset. Colors denote scLDM, Squidiff, CPA, and the linear additive baseline.

**Supplementary Figure. S3.**

**Supplementary Fig. S3. Per-combination comparison of R² on HVGs.** Each panel shows one fully held-out double-gene perturbation. Bars represent the R² on HVGs of scLDM, Squidiff, CPA, and the linear additive baseline for the corresponding held-out combination.

**Supplementary Figure. S4**.

**Supplementary Fig. S4. Comparison of mean expression PCC and delta PCC on HVGs across held-out combinations.** The upper panel shows mean expression PCC, which measures the agreement between predicted and observed average expression profiles. The lower panel shows delta PCC on HVGs, which measures the agreement between predicted and observed perturbation-induced expression changes on highly variable genes.

**Supplementary Figure. S5**.

**Supplementary Fig. S5. GO Biological Process enrichment analysis of true and predicted combinatorial perturbation responses.** For each held-out gene combination, differentially expressed genes were identified by comparing true perturbation versus control cells and predicted perturbation versus control cells, respectively. GO BP enrichment analysis was then performed using the top 100 DEGs from each comparison. Bubble plots show enriched GO BP terms for true responses, predicted responses, and side-by-side comparisons. Dot color indicates -log10(FDR), and dot size indicates the number of genes associated with each enriched term.

**Supplementary Tables**

**Supplementary Table S1. Evaluation of VAE reconstruction performance on the PBMC validation set.**

Reconstruction quality was evaluated using MSE, RMSE, MAE, and mean expression Pearson correlation coefficient (mean expression PCC). Metrics were calculated using all genes and the top 100 differentially expressed genes (DEGs).

| **Group** | **Feature set** | **Cells** | **MSE** | **RMSE** | **MAE** | **Mean expression PCC** |
| --- | --- | --- | --- | --- | --- | --- |
| Control | All genes | 765 | 0.0100 | 0.1001 | 0.0242 | 0.9833 |
| Control | Top 100 deg | 765 | 0.0164 | 0.1279 | 0.0478 | 0.9986 |
| Perturbed | All genes | 765 | 0.0094 | 0.0970 | 0.0231 | 0.9866 |
| Perturbed | Top 100 deg | 765 | 0.0402 | 0.2006 | 0.1219 | 0.9950 |
| All | All genes | 1530 | 0.0097 | 0.0985 | 0.0237 | 0.9856 |
| All | Top 100 deg | 1530 | 0.0283 | 0.1682 | 0.0848 | 0.9975 |

**Supplementary Table S2. Aggregate performance across 10 fully held-out double-gene perturbations.**

Mean and standard deviation of prediction performance across 10 fully held-out double-gene perturbations in the Norman Perturb-seq dataset. Models include scLDM, Squidiff, CPA, and the linear additive baseline. Metrics include R² on HVGs, mean expression PCC, delta PCC on HVGs, and DEG PCC.

| **Model** | **R² on HVGs** | **Mean expression PCC** | **Delta PCC on HVGs** | **DEG PCC** |
| --- | --- | --- | --- | --- |
| scLDM | 0.906 ± 0.025 | 0.966 ± 0.008 | 0.825 ± 0.047 | 0.873 ± 0.066 |
| Squidiff | 0.866 ± 0.070 | 0.954 ± 0.028 | 0.800 ± 0.075 | 0.872 ± 0.070 |
| CPA | 0.833 ± 0.066 | 0.942 ± 0.023 | 0.763 ± 0.085 | 0.866 ± 0.072 |
| Linear | 0.809 ± 0.073 | 0.945 ± 0.022 | 0.754 ± 0.081 | 0.864 ± 0.119 |
